# Multi-product calibration model for soluble solids and water content quantification in Cucurbitaceae family, using visible/near-infrared spectroscopy

**DOI:** 10.1016/j.heliyon.2021.e07677

**Published:** 2021-07-29

**Authors:** Kusumiyati, Yuda Hadiwijaya, Ine Elisa Putri, Agus Arip Munawar

**Affiliations:** a Department of Agronomy, Faculty of Agriculture, Universitas Padjadjaran, Sumedang 45363, Indonesia; b Department of Agricultural Engineering, Faculty of Agriculture, Universitas Syiah Kuala, Indonesia

**Keywords:** Data pre-processing, Prediction, Quality evaluation

## Abstract

Latest studies on Vis/NIR research mostly focused on particular products. Developing a model for a specific product is costly and laborious. This study utilized visible/near-infrared (Vis/NIR) spectroscopy to evaluate the quality attributes of six products of the Cucurbitaceae family, with a single estimation model, rather than individually. The study made use of six intact products, zucchini, bitter gourd, ridge gourd, melon, chayote, and cucumber. Subsequently, the multi-product models for soluble solids content (SSC) and water content were created using partial least squares regression (PLSR) method. The PLSR modeling produced satisfactory results, the coefficient of determination in calibration set (R^2^c) was discovered to be 0.95 and 0.92, while the root mean squares error of calibration (RMSEC) was found to be 0.41 and 0.61, for SSC and water content, respectively. These models were able to accurately predict the unknown samples with coefficient of determination in prediction set (R^2^p) of 0.96 and 0.92, as well as root mean squares error of prediction (RMSEP) of 0.32 and 0.58, while the ratio of prediction to deviation (RPD) was found to be 5.68 and 3.69 for SSC and water content, respectively. This shows Vis/NIR spectroscopy was able to quantify the SSC and water content of six products of Cucurbitaceae family, using a single model.

## 1 Introduction

Cucurbitaceae, often referred to as cucurbits, is the largest tropical vegetable group and is widely cultivated in various parts of the world. The cucurbits are consumed daily by numerous people, and are therefore regarded as essential food products. Nowadays, consumers are have a diverse option of products in the market. Therefore, products are expected to have high value, reliability and consistency. Meanwhile, the agricultural industry is faced with the challenge of examining food products and ensuring the demands of consumers are met. The inspection of fruits/vegetables involves internal constituents, including sugar content, water, and firmness, and external properties, including color, surface defects, shape, and size. However, the quality of agricultural goods is not fully assessable based on visuals, as this does not account for internal characteristics, including soluble solids content (SSC) and water content. These two attributes are very important aspects of consumers' acceptance of agricultural goods because both are associated with taste (Park et al., 2018). The measurement SSC and water content is generally performed through laboratory evaluation. For SSC, the standard method of measurement is the refractometric approach (Hadiwijaya et al., 2020b). This technique computes the refractive index of the juice sample using a refractometer. Meanwhile, conventional determination of water content is the sample drying method, using an oven, at a specific temperature and duration (Scalisi & O'Connell, 2020; Hadiwijaya et al., 2020a). Currently, the methods used to assess these food product properties seem time-consuming, and destructive, as products subjected to analyses are no longer sellable.

Several investigations have been conducted regarding non-destructive approaches to measure the quality parameters in agricultural goods, mostly fruits and vegetables, as an alternative to these conventional methods. These approaches include nuclear magnetic resonance (NMR) (Kamal et al., 2019), hyperspectral imaging (HSI) (Lu et al., 2017) and mid-infrared spectroscopy (Bureau et al., 2019). Non-destructive internal quality monitoring allows producers to provide the best product for consumers, and increases the product's selling value. In addition, this technique measures internal quality in a rapid and non-destructive manner. The HSI application for the SSC detection on kiwi fruit resulted in a coefficient of determination (R^2^) and ratio of prediction to deviation (RPD) of 0.95 and 3.12, respectively (Zhu et al., 2017). Another study successfully created an excellent calibration model for water content evaluation in apples using HSI (R^2^ = 1.00, root mean squares error (RMSE) = 0.04) (Crichton et al., 2017). Visible and near-infrared (Vis/NIR) spectroscopy is also a possible non-destructive, rapid, and precise technique for estimating the internal quality of various products. The Vis/NIR region (380–1050 nm) is promising as this is usually attributed to the 3rd and 4th overtones of O–H and C–H bands in sugar molecules (Cen and He, 2007). Numerous studies on Vis/NIR application in SSC quantification have also been conducted on citrus fruits (P. Li et al., 2020; Song et al., 2019), apple (Fan et al., 2020; Lan et al., 2021; Xia et al., 2020), pear (Mishra et al., 2021), melon (M. Li et al., 2019), grape (Fernández-Novales et al., 2019) and tomato (Huang et al., 2018). This technique was also able to accurately predict water content in pomelo (Xu et al., 2020), dates (Alhamdan and Atia, 2017), plum (Mulisa Bobasa et al., 2020; Posom et al., 2020), maize seed (Zhang and Guo, 2020) and olives (Lee et al., 2018).

The recently published studies on Vis/NIR research are about specific products. Creating a model for particular product is expensive and time-consuming. A possible solution involves collecting a diverse spectra dataset in a bid to produce one unique pattern for diverse applications in various products. Several researches have been conducted on the development of global multivariate models for rapid quality evaluation. In one study, an investigation was conducted to determine water content in freeze-dried drug products using NIR (Clavaud et al., 2017), while another study utilized NIR spectroscopy was utilized to build global models for the assessment of amylose, cellulose, and starch content in various tuber and root products (Masithoh et al., 2020). However, no research has been conducted on multi-product prediction based on Vis/NIR, in the Cucurbitaceae family. This study therefore aimed to develop a multi-product and Vis/NIR-based calibration model in a bid to quantify the SSC and water content of six products in the Cucurbitaceae family, zucchini, bitter gourd, ridge gourd, melon, chayote, and cucumber.

## 2 Materials and methods

### 2.1 Sample collection

The samples included six intact products in the Cucurbitaceae family, zucchini var. Jacky z-6, bitter gourd var. Hokian, ridge gourd var. Primavera, melon var. Mekarsari sh-1, cucumber var. Wulan, and a local variety of chayote. The melon and ridge gourd samples were harvested from potential product orchards in Sumedang, while the zucchini, bitter gourd, chayote, and cucumber samples were obtained from Bandung, Indonesia. These samples were collected at the harvesting stage, packed into fruit-plastic-baskets, and transported to the Horticulture Laboratory, Universitas Padjadjaran. The harvesting stage was determined based on the optimum maturity level usually harvested by farmers. At same day, the analysis of the samples was carried out (acquisition of spectra and reference data). Each product was represented by 50 samples. Therefore, a total of 300 samples were used in this research. The samples were cleaned and numbered prior to the analysis, and 200 were grouped into the calibration set, while the prediction set consisted of the remaining 100 samples.

### 2.2 Instrument

**Figure 1:**
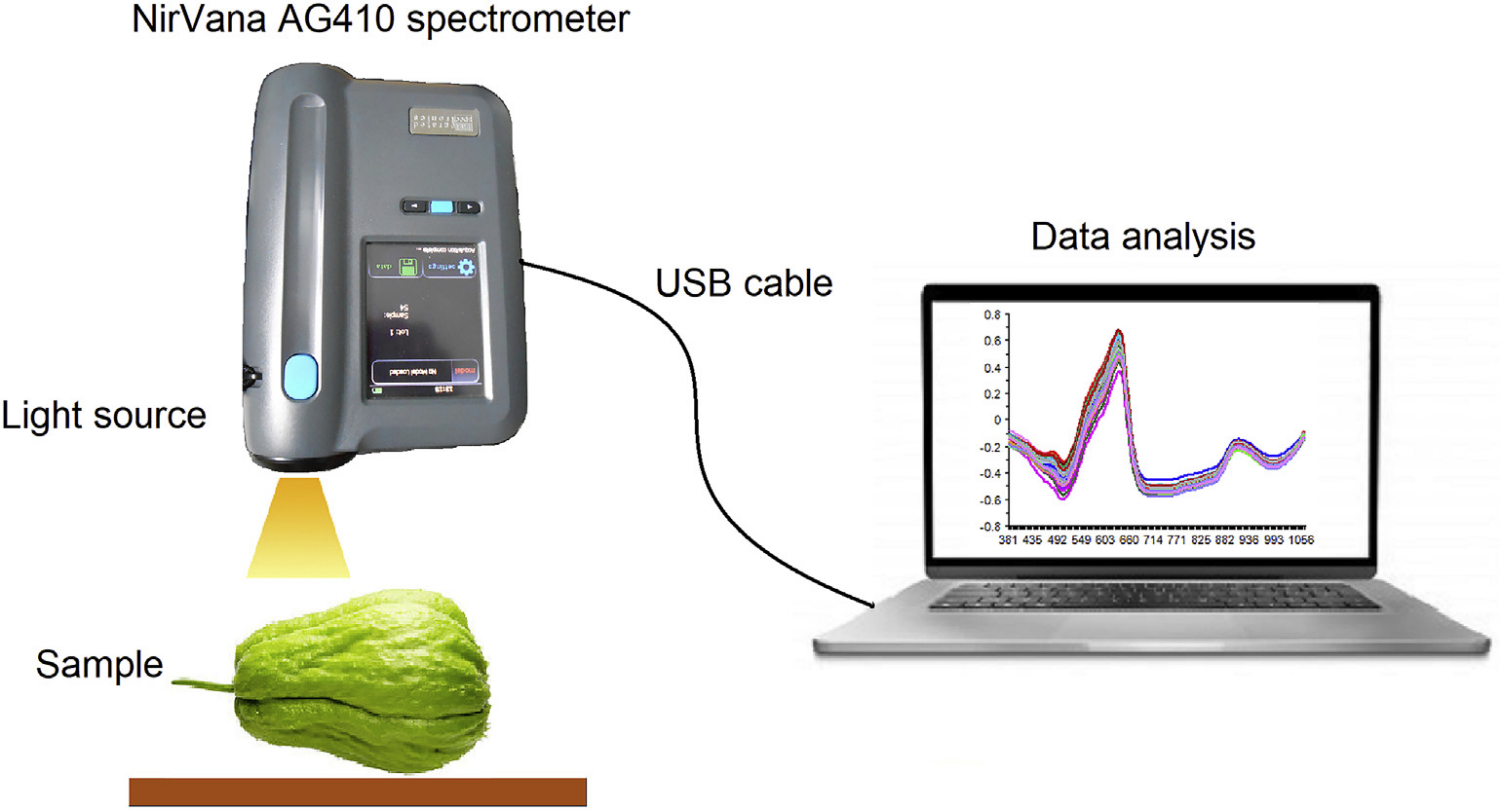
Illustration of the data acquisition.

Vis/NIR spectra were obtained using a NirVana AG410 (Integrated spectronics Pty, Ltd, Australia) spectrometer in the diffuse reflectance mode, and this was then automatically transformed into absorbance. Each spectrum contains sample absorbance at a wavelength between 381 and 1065 nm (3 nm pixel spacing and spectra resolution of 8–13 nm) for a total of 229 data points per spectrum. Subsequently, the absorbance spectra were captured by a vertical scan in the both opposite sides of the upper, middle, and lower part of sample. The sample absorbance data is calculated as the average of the six measurements per samples. Meanwhile, spectra collection was performed in the laboratory at room temperature (26 °C). The recorded spectra were then transferred to a computer for further analysis, while the spectra acquisition and actual data of SSC as well as water content evaluations were conducted on the same day. Figure 1 shows the illustration of spectra collection.

### 2.3 Determination of soluble solid content and water content

After spectra acquisition, the sample was cut across the circumference at the scanned area. Subsequently, SSC analysis was done on extracted samples and acquired using a digital refractometer (Atago, Japan) to enable %Brix measurement. The sample SSC was then evaluated based on the average value of three consecutive observations. Meanwhile, for the water content assessment, 30 g of sliced sample were placed on a small glass container. The water content was determined by oven-drying the sample at 60 °C, and weighing until a constant weight was achieved, and was calculated based on the percentage reduction in the sample weight due to drying.

### 2.4 Data analysis

Spectra analysis was completed using Microsoft Excel 2019 and The Unscrambler X 10.4 (trial version). These are appropriate for spectra interpretation, developing calibration models, and predicting independent samples. Meanwhile, spectra preprocessing was performed to reduce the effects of noise, scattering, and background interference. The original spectral data, as well as data previously processed using standard normal variate (SNV), moving average (MA), savitzky-golay smoothing (SGS), area normalization (AN), mean normalization (MN), first derivative (dg1) and second derivative (dg2) savitzky-golay, de-trending (DT), as well as orthogonal signal correction (OSC), were employed for modeling and evaluated to increase the regression result's efficiency. In addition, the principal component analysis (PCA) is used to eliminate correlated spectra variables, giving rise to new, simple and uncorrelated variables, and to visualize the spectra information. The model was calibrated using a partial least squares regression (PLSR) method, and validated using cross-validation technique. Cross-validation is useful for calculating the optimum PCs and avoiding overfitting in the calibration model. The datasets were split into two groups, the calibration and prediction set, as stated above. Each set comprised adequately distributed datasets representing the entire the SSC and water content varieties. The prediction set was designed to evaluate the predictive ability of a calibration model in determining the unknown samples. Subsequently, the model output was reported for the coefficient of determination in calibration (R^2^c), cross-validation (R^2^cv), and prediction (R^2^p), as well as the root mean square error of calibration (RMSEC), cross-validation (RMSECV), and prediction (RMSEP), and the ratio of prediction to deviation (RPD). The acceptable models ought to provide lower error, higher R^2^c, and RPD, but a slight gap in RMSEC and RMSEP (Khoshnoudi-Nia and Moosavi-Nasab, 2019; Cheng and Sun, 2015). Furthermore, only a few principal components (PCs) are also desirable in selecting the best calibration model.

## 3 Results and discussion

### 3.1 Analysis of reference data

Table 1 shows the descriptive actual data statistics of six products for each quality attributes. The SSC is assumed to be the constituent for the assessment of sugar content in agricultural products (Hadiwijaya et al., 2020b). In addition, the water content in food is also an important attribute because of the relation to the product's physical and chemical characteristics of the product, and the effect on food shelf-life (Hadiwijaya et al., 2020a). According to the data obtained, cucumbers had the lowest SSC mean value (3.17 %Brix), while chayote samples (89.80%) had the lowest mean water content. Meanwhile, the melon samples were discovered to have the highest mean SSC (8.48 %Brix) and water content (95.76%). The multi-product samples showed an extensive range in both attributes, and the distribution peak between 3.17 – 8.48 %Brix (SSC) and 89.80–95.76 % (water content) was due to the broad variation in the samples used. This is in line with the report by Rambo et al. (2016) stating various products ought to be combined in order to obtain a large range of chemical composition values in a single calibration model.

**Table 1:** Mean of SSC and water content data of samples being analyzed.

Samples	SSC (%Brix)	Water content (%)
Zucchini	3.76	93.99
Bitter gourd	4.05	90.28
Ridge gourd	3.23	92.16
Melon	8.48	95.76
Chayote	3.81	89.80
Cucumber	3.17	91.76
Combined data	4.42	92.29

SSC: soluble solids content.

### 3.2 Spectral features

**Figure 2:**
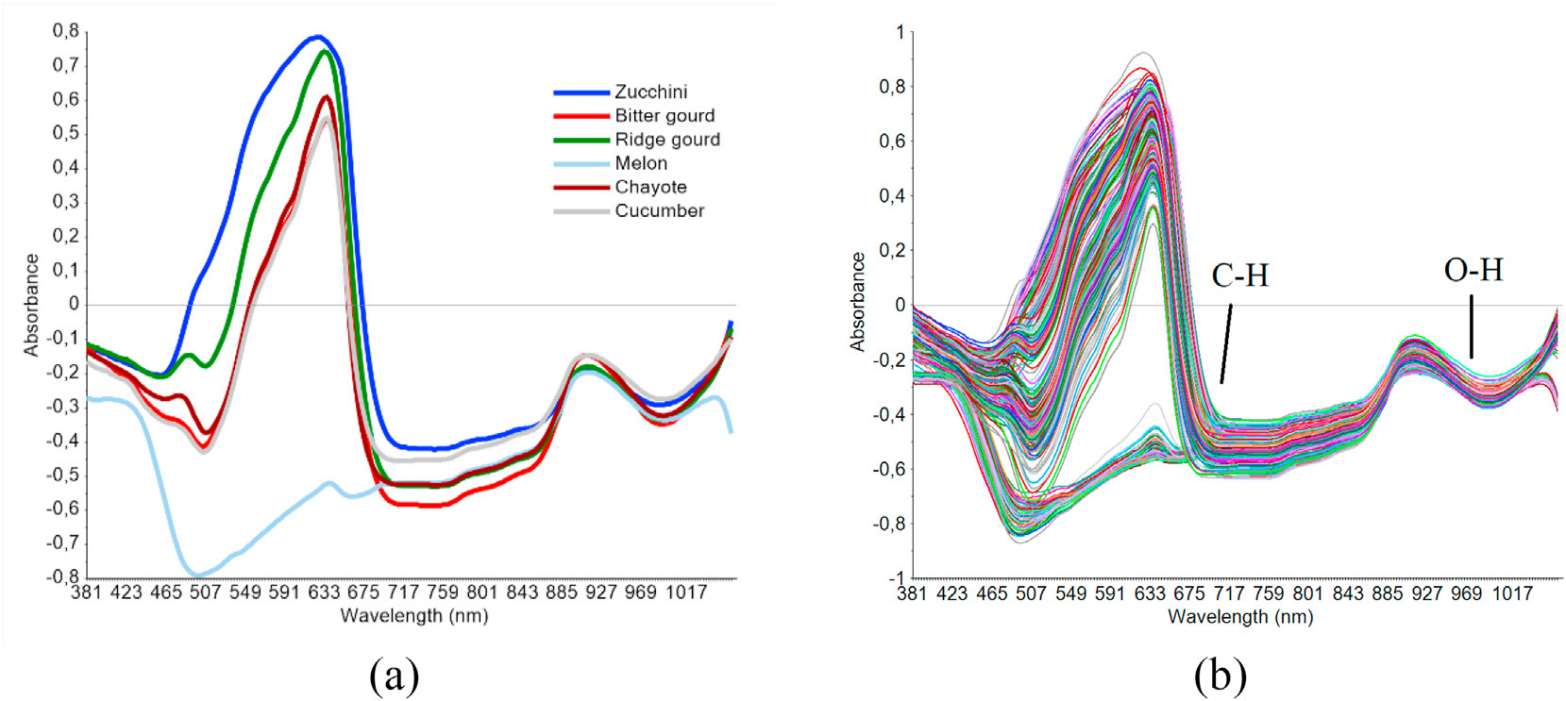
Original spectra data of samples (a) Example of spectrum of each product (b) All acquisitions.

Figure 2 (a) is an example spectrum for each product, while Figure 2 (b) shows the absorbance spectra of the all acquisitions. The Vis/NIR spectra obtained in this study had a similar pattern with the tomato absorbance data reported by Acharya et al. (2017). These sample spectra were relevant to fruit components involving the response of C–H and O–H molecular bonds. The samples belong to the same family and have many similarities, however, the six products possessed different spectra properties. This is possibly due to the differences in sample surface roughness, skin thickness, color, and chemical composition. The variation in absorbance within the visible region (381–700 nm) is mainly due to sample color. A wide peak was observed in each spectrum, around 630 nm, due to the absorption of chlorophyll (Zhang et al., 2019a). This peak was more prominent in the spectra of the green-skinned zucchini, bitter gourd, ridge gourd, chayote, and cucumber samples, compared to the yellow melon samples. A study by Zhang et al. (2020) described the valley in the spectrum was correlated with the C–H band's 4th overtone at 700 nm, and is useful for predicting fruit SSC. Meanwhile, the spectra absorbance at approximately 970 nm represented the O–H second overtone of carbohydrates and water (Guo et al., 2015). Information about the sample spectra's peaks and valleys are crucial in the modeling stage for either classification or forecasting. In this case, the chemometrics played a significant role in the spectral interpretation, including spectra preprocessing, PCA, and PLSR modeling.

**Figure 3:**
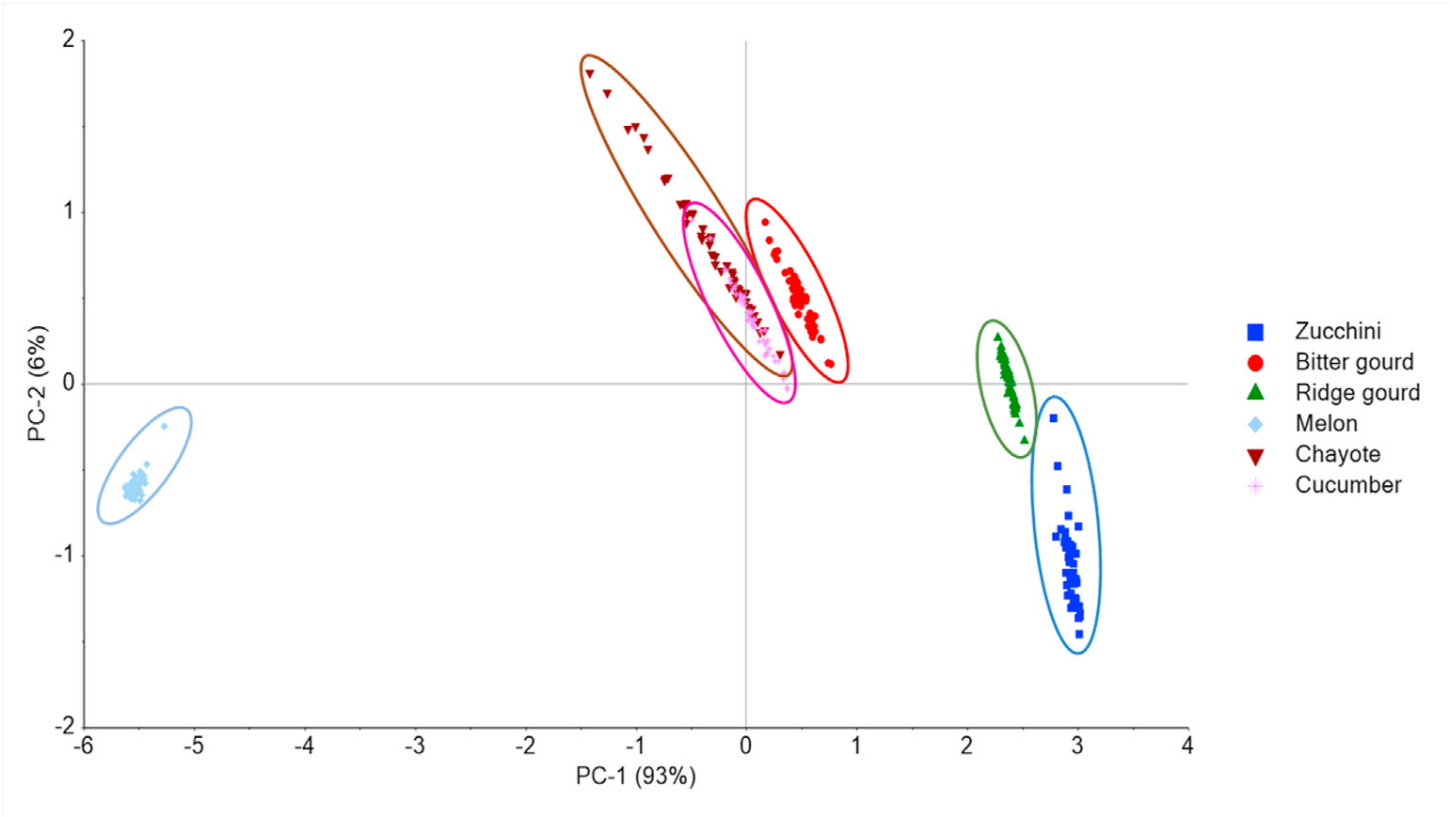
PCA scores plot extracted from the spectra data of multi-products samples.

The PCA offers insight on how explored spectra cause specific samples to differ or be identical. In this study, the dataset was composed of 300 observations (50 samples x 6 products) x 229 spectra variables. Prior to the PCA computation, MSC was applied to reduce the interference effect in the original spectra. Thus, for each sample, the light scattering of diffuse reflectance spectroscopy was approximated and adjusted to the ideal selected sample by averaging the spectra data (Rinnan et al., 2009). Figure 3 shows the plot of PCA scores obtained using the first and two components (PC1 and PC2), extracted from the original spectra coupled with MSC. PC1 and PC2 compensated for 99 % of the spectral variation. The PCA scores indicated the presence of several different products (although of the same family) influenced the features of the Vis/NIR spectra. Furthermore, the samples are classified into three perceivable groups. The first group comprised zucchini and ridge gourd, the second consisted of bitter gourd, chayote and cucumber, while the third comprised melon samples. From the PCA scores plot, combining different products into a single model was observed to lead to broad spectral variations. This is in line with the study by Zhang et al. (2019b), on the inspection of eight different apple cultivars, producing wide-ranging spectra.

### 3.3 Development of calibration models for SSC and water content

Prior to further modeling, several methods were selected to improve the original spectra. The spectra preprocessing stage was an essential aspect of multivariate calibration, majorly aimed at eliminating irrelevant background signal or distortion in the original spectra, in a bid to enhance predictive accuracy or data interpretation, thus, maximizing the correlation to the desired quality parameters (Schoot et al., 2020; Singpoonga et al., 2020) Meanwhile, PLSR was used to correlate the independent variables (absorbance spectra) with the dependent variables, the SSC and water content of intact zucchini, bitter gourd, ridge gourd, melon, chayote, cucumber (desired quality parameters). A single model was adopted for to evaluate the SSC of all samples, while another calibration model was employed to estimate the water content. The PLSR model development was completed using the full original and preprocessed spectra between 381 and 1065 nm. Table 2 shows the PLS analysis summary, describing PCs, R^2^c, R^2^cv, R^2^p, RMSEC, RMSECV, RMSEP, and RPD. The most suitable SSC model was determined by applying the OSC with R^2^c of 0.95, RMSEC of 0.32, and PCs of 1. In a bid to examine the effectiveness and reliability of the established model, the model was tested to predict the unknown samples (prediction set), and this resulted in an R^2^p of 0.96, RMSEP of 0.32, and RPD of 5.68. Meanwhile, in the water content evaluation, OSC was also appointed to correct the original spectra, thus producing the optimum model (R^2^c = 0.92, RMSEC = 0.61, and PCs = 1), able to predict the water content with satisfactory results (R^2^p = 0.92, RMSEP = 0.58, and RPD = 3.69). Rambo et al. (2016) investigated a broad dataset model covering coconut, banana, and coffee biomasses, using a spectra range of 400–2500 nm. The calibration model of the quality parameters associated with this study, obtained satisfactory results, with water content and total sugar R^2^c = 0.82 and 0.87, and RPD = 7.40 and 12.13, respectively. In another study by Clavaud et al. (2017) on the development of a global model, NIR (4000–10000 cm^−1^) was used to quantify the moisture content in various freeze-dried medicines. Satisfactory correlation between spectra and moisture content reference data was indicated by an R^2^c of 0.97 and RPD of 6. Masithoh et al. (2020) also conducted multi-product modeling with a large dataset, to determine the polysaccharide content. The maximum R^2^c and RPD obtained in starch content assessment, were 0.95 and 4.47. This is in line with the current research, as the R^2^c and RPD calculated were above 0.80 and 3.00, respectively, indicating the multi-products model yielded excellent performance.

**Table 2:** The statistical results of multi-products models for soluble solids content and water content determination.

Quality attribute	Spectra preprocessing	PCs	R^2^c	R^2^cv	R^2^p	RMSEC	RMSECV	RMSEP	RPD
Soluble Solids Content	Original	3	0.92	0.91	0.94	0.54	0.55	0.43	4.24
	SNV	3	0.91	0.91	0.93	0.56	0.58	0.47	3.95
	MA	3	0.92	0.91	0.94	0.54	0.56	0.43	4.24
	SGS	3	0.92	0.91	0.94	0.54	0.55	0.43	4.24
	AN	10	0.91	0.89	0.94	0.56	0.65	0.45	4.11
	MN	10	0.91	0.88	0.94	0.56	0.65	0.45	4.11
	dg1	3	0.92	0.92	0.94	0.52	0.54	0.41	4.45
	dg2	4	0.93	0.92	0.94	0.50	0.53	0.43	4.30
	DT	3	0.92	0.92	0.95	0.52	0.54	0.40	4.58
	OSC	1	0.95	0.95	0.96	0.41	0.42	0.32	5.68
Water Content	Original	5	0.88	0.87	0.86	0.76	0.78	0.78	2.73
	SNV	8	0.90	0.89	0.91	0.67	0.72	0.61	3.51
	MA	5	0.88	0.88	0.87	0.74	0.77	0.76	2.80
	SGS	5	0.88	0.88	0.86	0.76	0.76	0.78	2.73
	AN	6	0.88	0.86	0.84	0.76	0.81	0.83	2.55
	MN	6	0.88	0.86	0.84	0.76	0.81	0.83	2.55
	dg1	7	0.90	0.89	0.91	0.67	0.73	0.61	3.49
	dg2	5	0.89	0.88	0.92	0.71	0.76	0.59	3.61
	DT	4	0.87	0.86	0.87	0.80	0.83	0.76	2.82
	OSC	1	0.92	0.92	0.92	0.61	0.61	0.58	3.69

SNV: standard normal variate, MA: moving average, SGS: savitzky-golay smoothing, AN: area normalization, MN: mean normalization, dg1: first derivative savitzky-golay, dg2: second derivative savitzky-golay, DT: de-trending, OSC: orthogonal signal correction, PCs: principal components, R^2^c: coefficient of determination in calibration set, R^2^cv: coefficient of determination of cross-validation, R^2^p: coefficient of determination in prediction set, RMSEC: root mean square error of calibration, RMSECV: root mean square error of cross-validation, RMSEP: root mean square error of prediction, RPD: the ratio of prediction to deviation.

**Figure 4:**
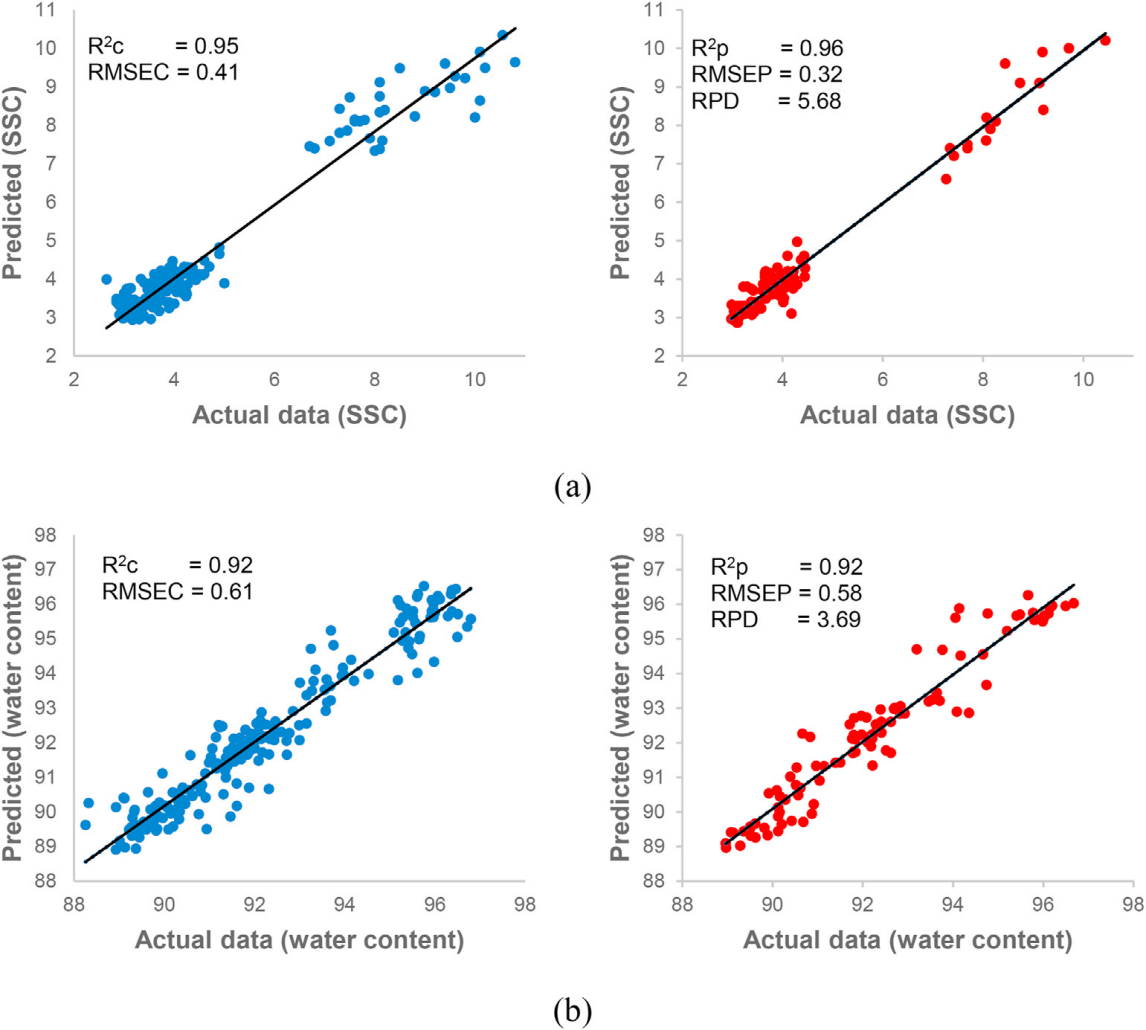
Scatter plot of multi-products calibration set (blue) and prediction set (red) for SSC (a) and water content (b), developed using PLSR and OSC.

The high accuracy of the model attained in this study, was due to the broad datasets used. Large data variability in spectroscopic techniques possibly enhances the model robustness. Therefore, the results obtained were better, compared to the report by Li et al. (2019), estimating SSC in melons with R^2^c and RPD of 0.83 and 2.39, respectively. Basically, all the calibration models listed in Table 2 are able to determine SSC and water content. However, the model generated from the OSC spectra is most suitable for application in the agricultural industry, due to performance, as the OSC minimizes any variables in the original spectra unorthogonally associated with the desired quality parameters. This is in line with the previous research by Hemrattrakun et al. (2020) on the use of Vis/NIR and OSC to detect SSC, ascorbic acid, and firmness in persimmon fruit (correlation coefficient (R) = 0.86, 0.89, and 0.87, respectively). The results of the conventional method of assessment was graphed against the Vis/NIR observation counterpart, to provide a detailed model reliability image. Figure 4 shows the scatter plots of calibration and prediction sets for SSC and water content. The sample appears to be scattered throughout the regression line in both groups, indicating the Vis/NIR prediction did not differ from the measured water content and SSC. This confirmed the calibration models in this experiment were suitable for further application (Nicolaï et al., 2007). Therefore, the Vis/NIR technique was concluded to be a reliable replacement for the conventional methods of quality evaluation in agricultural products. However, the developed models in this study were discovered to have a few limitations. These models are applicable to the products previously calibrated. This means a different product must be included in the calibration set and regression modeling, prior to assessment.

### 3.4 Regression coefficient and sensitive wavelengths

**Figure 5:**
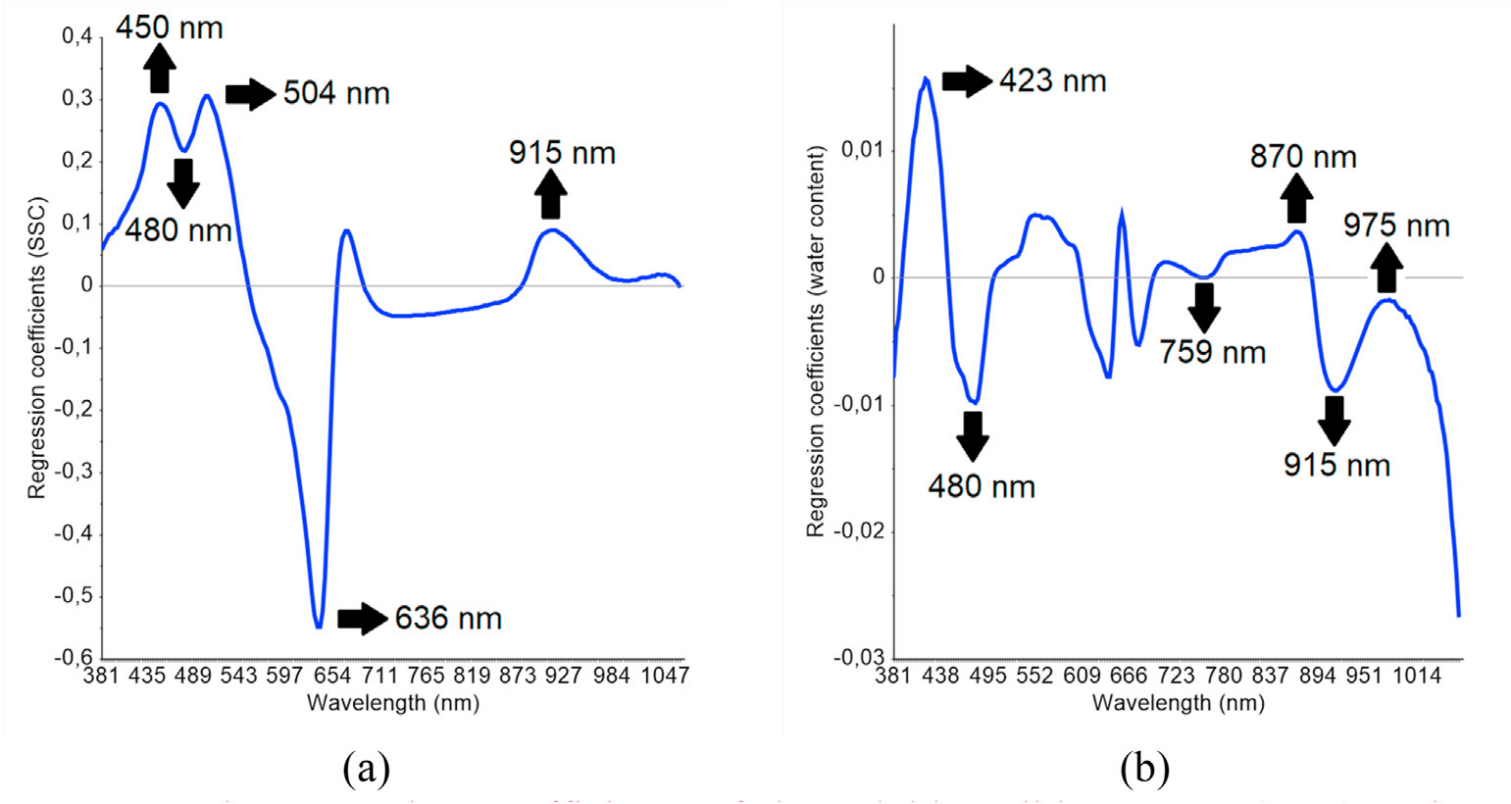
The regression coefficients of the soluble solids content (SSC) and water content multi-products calibration models.

Figure 5 shows the regression coefficient (RC) for predicting SSC (a) and water content (b) of six products, zucchini, bitter gourd, ridge gourd, melon, chayote, and cucumber. The RC on the PLSR calibration model defines the bands correlated with SSC and water content. Furthermore, each wavelength with a high RC (positive/negative) peak has a significant impact on model development. Table 3 shows the absorption band data derived from the RC plots. The RCs both display different trends, however, the two quality parameters were discovered to have similar peaks, specifically at 915 nm, and this correlates with the third overtone of the C–H band. This waveband is characteristic of carbohydrates, and is defined as the main structure of starch content (Maraphum et al., 2020). In addition, identical sharp peaks were observed around 480 nm for both quality parameters. Absorption peaks characteristic of starch content were also found at 423, 450, 480, and 504 nm. Meanwhile, SSC related peaks were discovered at 636 nm, and this was the amylose waveband (Bantadjan et al., 2020b). The RC peaks for water content appeared at 759 nm (third overtone of the O–H band) and 975 nm (second overtone of the O–H band), corresponding to water molecules (Phetpan et al., 2018; Posom et al., 2020; Bantadjan et al., 2020a). This confirmed the SSC prediction was influenced by the C–H band, while the water content determination referred to the bond vibration of O–H and C–H.

**Table 3:** The absorption bands identified from the regression coefficient plot in the multi-products calibration models of SSC and water content.

Quality attribute	Sensitive wavelength (nm)	Wavelength (nm) reported from other studies	Bond vibration	Structure
SSC	450, 480, and 504	443 and 490 (Bantadjan et al., 2020b)		Starch
	636	635 (Ziba et al., 2019)		Starch, amylose
	915	890 (Phetpan et al., 2018) 873, 910, and 913 (Posom et al., 2020; Maraphum et al., 2020; Phuphaphud et al., 2020)	3^rd^ overtone of band C–H	Carbohydrates, starch
Water content	423, 480	443 and 490 (Bantadjan et al., 2020b)		Starch
	759	755 (Phetpan et al., 2018)	3^rd^ overtone of band O–H	Water
	870	890 (Phetpan et al., 2018)	3^rd^ overtone of band C–H	Carbohydrates, starch
	915	910 and 913 (Posom et al., 2020; Maraphum et al., 2020; Phuphaphud et al., 2020)		
	975	970-975 (Posom et al., 2020; Bantadjan et al., 2020a)	2^nd^ overtone of band O–H	Water

SSC: soluble solids content.

## 4 Conclusions

This experiment showed Vis/NIR spectroscopy is able to quantify SSC and water content in six fruits and vegetables of the Cucurbitaceae family, including zucchini, bitter gourd, ridge gourd, melon, chayote, and cucumber. The multi-products models obtained R^2^c and RMSEC values of 0.95 and 0.41 for SSC, as well as 0.92, and 0.61 for water content. Subsequently, the calibration model was used to estimate different samples, and R^2^p of 0.96 and 0.92, RMSEC of 0.32, and 0.58, as well as RPD of 5.68 and 3.69 were obtained for SSC and water content, respectively. These statistical calculations confirm the development of multi-product calibration models is suitable for the prediction of SSC and water content in members of the Cucurbitaceae family.

The idea of lead time reduction is still a concern in the agricultural sector. As a result, innovative technologies are used to expedite the quality control of foods. The potential to use a single model across multiple products to perform a rapid and non-destructive evaluation of SSC and water content represent a significant advancement in the analysis of in-lab samples. Therefore, development of multi-product models are time and cost-effective, compared to the single product analysis.

## Declarations

### Author contribution statement

Kusumiyati: Conceived and designed the experiments; Performed the experiments; Analyzed and interpreted the data; Contributed reagents, materials, analysis tools or data; Wrote the paper.

Yuda Hadiwijaya, Ine Elisa Putri & Agus Arip Munawar: Performed the experiments; Analyzed and interpreted the data.

### Funding statement

This research did not receive any specific grant from funding agencies in the public, commercial, or not-for-profit sectors.

### Data availability statement

Data included in article/supp. material/referenced in article.

### Declaration of interests statement

The authors declare no conflict of interest.

### Additional information

No additional information is available for this paper.
